# On the Influence of Flame-Retardant Additives on UV-Curable Thermosetting Glass Fiber-Reinforced Composites

**DOI:** 10.3390/polym15010240

**Published:** 2023-01-03

**Authors:** Natalia Gutiérrez Pérez-de-Eulate, Patricia Ares Elejoste, Garazi Goenaga, Maitane Urrutxua, Francisco Javier Vallejo, Jesús Ballestero, Alexandra Allue, José Luis Gómez-Alonso

**Affiliations:** 1 IDEKO Technology Centre, Basque Research and Technology Alliance (BRTA), Arriaga Industrialdea 2, 20870 Elgoibar, Spain; 2 GAIKER Technology Centre, Basque Research and Technology Alliance (BRTA), Parque Tecnológico de Bizkaia, Edificio 202, 48170 Zamudio, Spain

**Keywords:** flame-retardant, aluminum trihydrate, cone calorimeter, ultraviolet curing, dielectric analysis

## Abstract

One of the main advantages of fiber-reinforced polymer (FRP) composites is the ability to reduce their weight while they exhibit exceptional properties such as high strength, stiffness, and resistance to corrosion, and reduction in their lifetime maintenance when they are compared to the metallic components. These features led fiber-reinforced polymer composites to have applications in the mechanical, construction, aerospace, automotive, medical, marine, and other manufacturing industries. However, the use of this type of material is not possible in all of these applications since, in certain sectors, the fire resistance property that the material must present is one of the key factors. For this reason, a thermosetting resin composed of ultraviolet (UV)-curable acrylic monomers has been used as a matrix, where transparent aluminum trihydrate (ATH) flame-retardant fillers were incorporated for manufacturing flame-retarded UV-curable composites. The composite parts were produced by using glass fiber-reinforced UV-curable prepregs. An exhaustive study of different types of ATH-based flame-retardant additives and the possible cooperation between them to improve the fire properties of the UV-curable composite was carried out. Additionally, the most suitable additive percentage to meet the railway sector requirements was also evaluated, as well as the evolution in the viscosity of the matrix and its processing capacity during the manufacture of the prepregs at 60 °C. The compatibility between the fillers and the matrix was assessed using a dielectric analysis (DEA). The fire properties of both the matrix and the final composite were established.

## 1. Introduction

Fiber-reinforced polymer (FRP) composites are distinguished from other materials such as metals and ceramics by their high specific strength and stiffness, lightness, good corrosion and fatigue resistance, and design flexibility. These key factors are responsible for the continuous increase in the use of FRP composites in multiple applications such as aircraft, automotive, transport, ship or storage tank production [1]. Regarding the railway sectors, there have been many innovative actions to advance the introduction of composite materials in structural and rolling stock parts [2]. However, in the current transport sector, the composite parts that are used in the indoor applications are manually manufactured. As a consequence, there is a need for automation at an affordable cost.

One way to achieve easier automation of the manufacturing process for glass fiber composites is the use of ultraviolet (UV) light in the polymerization of the resins. This permits the manufacturing of composites at a faster timescale. The resin reaction is carried out at room temperature using eco-friendly, compact, and relatively cheap irradiation devices. Currently, the UV light is applied to small and flat substrates such as polymeric coatings, printing inks, and adhesives, therefore, to ensure efficient curing, in-depth photo-initiators with wavelengths of activation that are between 360 and 405 nm should be used because of their ability to penetrate much deeper than that those which function at short wavelengths [3].

The main drawback of using polymeric-based composites is the high content of hydrocarbon in the resin, therefore, the risk of a fire occurring is high. According to the investigations that were followed in [4], the combustion process of polymeric materials can be divided into five phases: the heating of the sample, the decomposition and emission of flammable and non-flammable gases, the ignition and smouldering of the combustible gases, the flame spread (fire development), and its extinction. In order to counter this phenomenon, many kinds of flame-retardant (FR) compounds have been applied to improve the flame retardancy of unsaturated polyester resins. Some examples are metal hydroxides and halogen, phosphorous, nitrogen and boron-based compounds. Moreover, new-generation flame-retardants, such as nanoparticles, expandable graphite, and bio-based [5] flame-retardants are also now being used. Halogenated additives [4,6], despite their great effectiveness, are being restricted by new environmental directives such as REACH, WEEE, and RoHS due to their harmful effects on humans and the environment. Currently, the most effective alternative to halogenated additives are compounds based on phosphorus and nitrogen and inorganic compounds, such as ATH and magnesium hydroxide fillers, which act both as flame-retardants and smoke suppressants. These mineral filler fire retardants, besides being non-toxic, have the characteristic of being the hydrated oxide of the metal, and they decompose endothermically with the release of water to the vapour phase to dilute the fuel load in the combustion zone. Furthermore, they promote the formation of a protective layer at the surface of the degrading matrix to limit the heat feedback in the combustion zone. Moreover, it should be noted that to achieve the building and transport sector fire safety specifications, high concentrations of these additives are required [7]. This fact leads to more difficult processability for the composites. Specifically, fire behaviour in the railway industry is defined by standard EN 45545, which specifies the requirements and the composition of the materials and products that are used in rail vehicles. The standard procedure covers seven parts, and it arose as a European criteria unification for railway applications, with a focus on safety and fire protection in passenger rail vehicles. The purposes of the standard procedure are to reduce the risk of fire generation, control its development and extinction, as well as to minimize the damage that the products generated during the fire, mainly dense smoke and toxic gases, may cause to the passengers and crew.

Within the standard EN 45545, in part 2, 28 requirements are defined for materials that are used in each part of the rail vehicle (wall, floor, seat, cab housing, roof, etc.), the operational route (elevated structures, tunnels, underground, etc.), and the vehicle class (standard, double-decked, napping and couchette vehicles, and vehicles forming part of an automatic train that has no emergency trained staff onboard).

Regarding the standard procedure that is mentioned above, on the one hand, it can be said that Requirement 1 is the strictest requirement which can be demanded for the composite parts in a rail vehicle for indoor applications, and on the other hand, HL3 is the strictest hazard level that can be demanded regarding the operational category of the train and type of vehicle. 

Any large composite part that, after being tested according to the established regulations, meets the requirements for the classification R1HL3 may be used for any indoor application in any railway vehicle.

The composite matrix curing degree has been analyzed by dielectric analysis (DEA). The fiber content was determined by calcination, and the flammability properties measurements were assessed using cone calorimetry, as well as smoke density and gas toxicity and lateral flame propagation.

## 2. Materials and Methods

### 2.1. Materials

The manufactured UV composites were produced according to the previous publication [8] based on glass-fiber-reinforced acrylic polyester. The acrylic resin that was used was a UV-curable resin composed of two polyester oligomers (TES21100 and VTC50) and an acrylic monomer (VTC5), and these were supplied by IVM Chemicals, Parona, Italy. The photo-initiator was bis(2,4,6-trimethyl benzoyl) phosphine oxide (BAPO), and its trade name is Irgacure^®^ 819, which was supplied by BASF the Chemical Company, Barcelona, Spain. Regarding the reinforcements, three types of chopped strand mats with area weights of 225, 300, and 450 g/m^2^, respectively, and a combined fabric composed of woven material and chopped strand mat (500 g/m^2^), which were supplied by 3B-the Fiberglass Company, Hoeilaart, Belgium, were used. Each reinforcement was named MAT225, MAT300, MAT450, and COMBI500, respectively.

The flame-retardant fillers that were used were ATH, with the commercial names Apyral 20X, 30X, 22, and 33, which were kindly supplied by Nabaltec Schwandorf, Germany. Table 1 shows the main differences among the grades of Apyral such as the particle size (D90), the specific surface area (BET), and the oil absorption.

### 2.2. Preparation of Formulations

The formulations were composed of resin and flame-retardant fillers (included in the formulation by parts per hundred resin (phr) by weight), which were mixed by sonication at 60 °C for 15 min. The compositions of the prepared formulations depending on the content of the flame-retardant filler are detailed in Table 2. An unfilled formulation (Formulation_0) was prepared as a control sample according to a previous publication [8].

### 2.3. Fire Retardant Prepregs and Composites Manufacturing Processes

**Figure 1:**
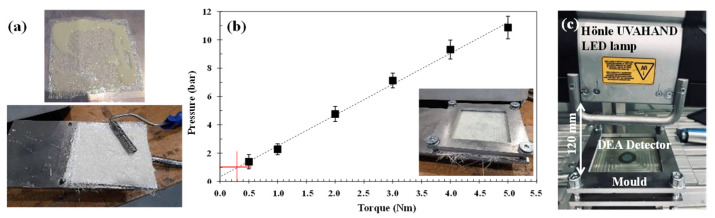
(**a**) Prepregs and composite manufacturing stages; (**b**) pressure applied in the final composite vs. the torque used to grip the tool. The standard deviation of the three samples is represented by the error bars. (**c**) Experimental DEA curing setup.

The prepregs manufacturing process was based on a previous publication [8], which consists of the impregnation of the fibers with UV-curable formulation, which was followed by a pre-curing process. Figure 1a shows the impregnation process, which was carried out using hand rollers. The pre-curing process was performed by using a Hönle UVAHAND LED lamp at a height of 120 mm from the sample, with a lamp intensity of 3% and a wavelength of 405 nm. Regarding the hand lay-up process, the four layers of reinforcement, namely, MAT225, MAT300, MAT450, and COMBI500, were placed in a flat mould, and the formulation was applied until the reinforcement was adequately impregnated. The composites were consolidated at 50% UV lamp intensity with a compaction pressure of 0.3 N.m, which corresponds to a pressure of 1 bar (Figure 1b).

### 2.4. Curing Degree Analysis

In the present study, the neat resin, the resin/flame-retardants filler blends, and the fire-retardant composites’ curing behaviour were monitored according to the evolution of the ionic viscosity over time [9], by means of DEA288 Epsilon analyser commercialised by NETZSCH. The samples were placed on the top of a coated tool mountable comb electrode (TMCc), as can be seen in Figure 1c.

### 2.5. Composite Fiber Content, Flexural, and Interlaminar Shear Strength (ILSS) Measurements

The glass fiber weight fraction of the composites was calculated according to the ASTM D3171-15 standard. The matrix was removed by heating the samples at 600 °C for 6 h in preheated crucibles. The reinforcements were weighed after cooling them to room temperature in a desiccator. The flexural properties were measured according to ASTM D790-10. The distance between the two supports was set at 40 mm, and the loading rate was 1 mm/min. The interlaminar shear strength (ILSS) was carried out following UNE EN ISO 14130:1999, with a loading rate of 1 mm/min. Both of the experiments were tested using the Shimadzu AG-X 5 kN universal testing equipment. The specimens were conditioned before testing for 200 h at 23 °C and 50% RH (relative humidity), and this was conducted at room temperature. The resulting values were obtained from the average of five specimens.

### 2.6. Fire Behaviour of Composite Parts Test Methods

The characterization of the composite parts was carried out according to EN 45545-2 standard, by us performing the necessary tests to assess whether the composite reaches the parameters that are demanded by R1 requirement of the standard and to the materials intended for the interior of an underground vehicle, which is the most restrictive hazard level that was collected (HL3).

The tests that were carried out are the following:

- A calorimetric cone was used according to the ISO 5660-1:2015 + A1:2019 standard to determine MARHE (Maximum Average Rate of Heat Emission) parameter.
- A determination of the optical density of smoke according to the ISO 5659-2:2017 standard and the toxicity of gases was performed under the EN 45545-2:2013 + A1:2015, Annex C, Method 1 standard to determine smoke density at the 4th minute, VOF4, the maximum density during the test, and the gas conventional toxicity indexes at the 4th and 8th minute parameters.
- A lateral flame propagation test was performed according to ISO 5658-2:2006 + AMD.1:2011 to determine the CFE (Critical Flux of Extinction) parameter.

#### 2.6.1. Cone Calorimeter

In the cone calorimeter test, which was governed by the standard ISO 5660-1, the 100 mm × 100 mm specimens were prepared and exposed to a controlled level of radiation to produce a combustion by an external spark. During this process, a radiant heating of 50 kW/m^2^ was used, and the gases generated were collected using a hood. In order to determine the heat release rate (HRR), oxygen consumption measurements were made by collecting the data on the flow rate in the exhaust duct and oxygen concentration at 2 s intervals. The HRR was used to calculate the ARHE curve (Average Rate of Heat Emission), which maximum is the MARHE parameter (Maximum Average Rate of Heat Emission) that is used for classification purposes.

#### 2.6.2. Smoke and Gas Production

In the test method that is described in the standard ISO 5659-2 specifications, specimens of 75 mm × 75 mm were exposed to controlled levels of radiant heating, in a similar way as they were in ISO 5660-1, by introducing them in a 0.5 m^3^ sealed chamber and positioning them horizontally and covering the sample in a retainer frame. The tests were carried out at a 50 kW/m^2^ irradiance level, without the application of a pilot flame. The tests lasted 10 min for classification purposes according to EN 45545-2. 

Using the light beam of the photometric equipment which was placed vertically in the sealed chamber, the specific optical density of the smoke released by the specimen was measured every 3 s.

The gas toxicity was determined by an FTIR gas analysis and according to the EN 45545-2:2013 + A1:2015, Annex C, Method 1 standard. A small gas sample was taken from the geometrical centre of the test chamber at the 4th and 8th minutes and for 30 s. Later, the sample was passed through an infrared spectrometer to quantify the concentrations of the CO, CO_2_, NO, NO_2_, SO_2_, HCN, HF, HCl, and HBr gases emitted by the tested specimen during the test.

According to EN 45545-2 for fire safety on trains, the following parameters must be determined: the smoke optical density at minute 4 (Ds 4min), the accumulated heat of the specific optical densities in the first 4 min of the test (VOF4), and the maximum smoke optical density (Ds max). Regarding the gas toxicity parameters, the Conventional Gas Toxicity Index (CITG) was determined at minutes 4 and 8 for each toxic gas that was measured. 

#### 2.6.3. Lateral Flame Propagation

To determine the fire behaviour when a flame front is observed on a vertical surface, it is necessary to use the international standard ISO 5658-2 according to the system for fire safety railway vehicles. For this purpose, the test specimens were placed in a vertical position and subjected to radiant heat in a radiant panel that was fed with methane. Once the specimen had been placed, the action of a small flame acted as an ignition front, so that the maximum distance travelled by the flame front on the surface of the specimen during the test could be observed. 

The above-mentioned distance was used to calculate the CFE (Critical Flux at Extinction) in kW/m^2^, which is defined as the heat flux incident on the surface of the specimen at the point where the flame front ceases. To do this, the maximum flame spread was measured, and this value is related to the corresponding heat flux value obtained from the calibration curve, which is based on measurements that were carried out using a heat flux meter at different positions on a non-combustible board.

## 3. Results and Discussion

### 3.1. Curing Degree of Resin/Flame-Retardant Filler Formulations and Composites

#### 3.1.1. Resin/Flame-Retardant Fillers Formulations

**Figure 2:**
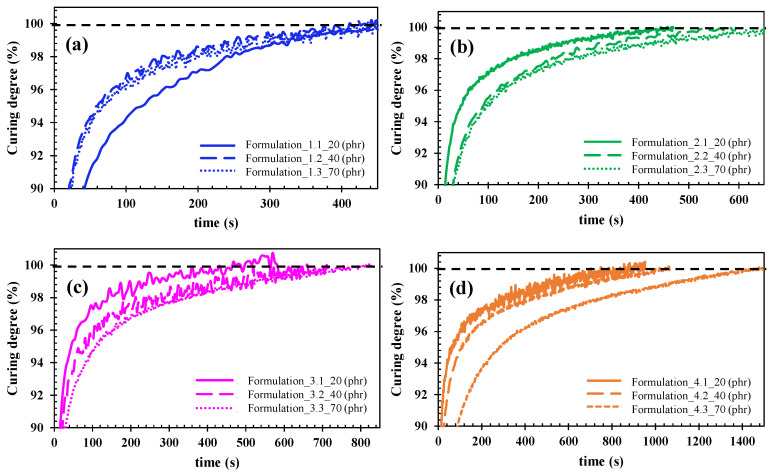
Curing degree evolution for all resin/flame-retardant fillers formulations as a function of time. (**a**) Formulation 1 (fillers Apyral20X at 20 (1.1), 40 (1.2) and 70 (1.3) phr); (**b**) Formulation 2 (fillers Apyral 30X at 20 (2.1), 40 (2.2) and 70 (2.3) phr); (**c**) Formulation 3 (fillers Apyral 22 at 20 (3.1), 40 (3.2) and 70 (3.3) phr); (**d**) Formulation 4 (fillers Apyral 33 at 20 (4.1), 40 (4.2) and 70 (4.3) phr). Dotted lines are drawn for visual interpretation of total curing degree.

Figure 2 shows the magnification of the curing behaviour for the four types of fire-retardant fillers depending on their content in the UV resin system as a function of time. The maximum conversion is displayed for when the ionic viscosity curves reached the 100% curing degree, which is represented by the dashed lines.

It is observed that the total curing degree, which takes place at the plateau of the curve, differs as a function of ATH grade and content. Using Figure 2, the different plateau times were extracted, and they are summarized in Table 3.

**Figure 3:**
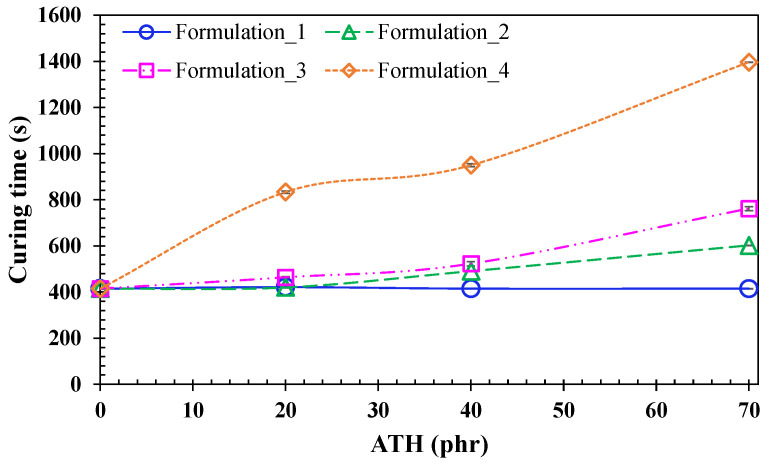
Curing degree behavior for the four groups of formulation as a function of the amount of filler in the neat UV resin system. Depicted lines are for visual interpretation.

Figure 3 shows the plateau values that are summarized in Table 3 over a range from 400 to 1400 s.

By analysing the curves, it can be stated that the light transmittance is reduced due to the presence of ATH in the case of formulations 2, 3, and 4, so the curing time is increased. However, formulation 1 (Apyral20X filler grade) remains constant despite the increasing ATH content in the resin formulation. The main differences between formulations 1 and 2, 3, and 4 are the lower specific surface area of the Apyral20X sets in 1.2 m^2^/g, and a higher D90 thereof. According to other investigations [10], large particle size fillers can reduce the scattering of the UV radiation, leading to the improvement of the curing depths in a composite system, which is noticeable as larger-sized fillers facilitate the UV resin curing behaviour. Fillers with higher specific surface areas tend to agglomerate, causing a strong increase in the viscosity of the system, therefore, reducing the mobility of the molecules [11]. Furthermore, in systems with higher specific surface areas, there is more absorption of the hardener and initiator, so a higher fraction of those are inactive in the matrix phase, which leads to a decelerated curing speed. Hence, from a manufacturing point of view, the most suitable formulation for producing glass fiber composites will be formulation 1 which is made of Apyral20X, with a lower specific surface area, which shows an invariable curing behaviour, regardless of the ATH content.

**Figure 4:**
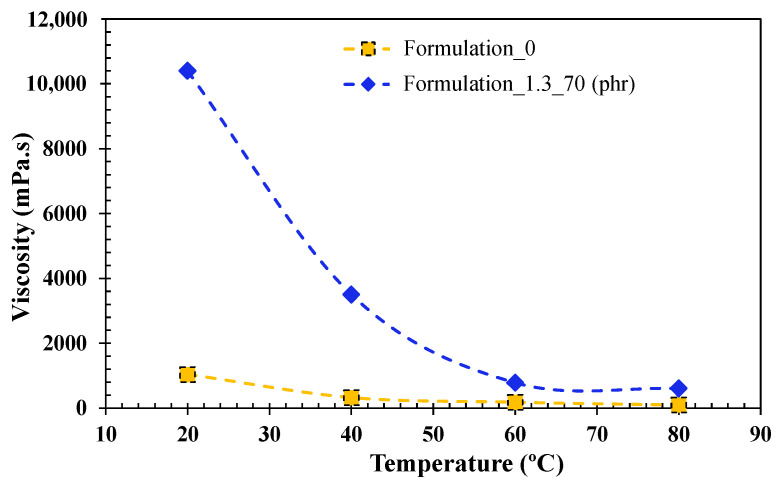
Viscosity profile of UV resin and formulation 1.3 with 70 (phr) as a function of temperature. Depicted dashes are for visual interpretation.

Figure 4 shows the viscosity profile for the neat UV resin (formulation 0) and formulation 1.3 with 70 phr of ATH. The representation of both systems shows that formulation 1.3 has to be processed at 60 °C in order to achieve an appropriate impregnation.

#### 3.1.2. Fire-Retardant Composite Manufacturing Results

Table 4 shows the composition of the manufactured UV-curable composite parts. It was made using reinforcements with different surface densities (225, 300, 450, and 500 g/m^2^) and different formulations as a matrix. It has to be noted that for the manufacturing of the fire-retarded composite, formulation 1.3 was selected for the reasons that are mentioned in Section 3.1.1. For this reason and in order to fulfil the specifications needed for the indoor parts in underground vehicles (R1HL3), composite 1.3 was manufactured.

**Figure 5:**
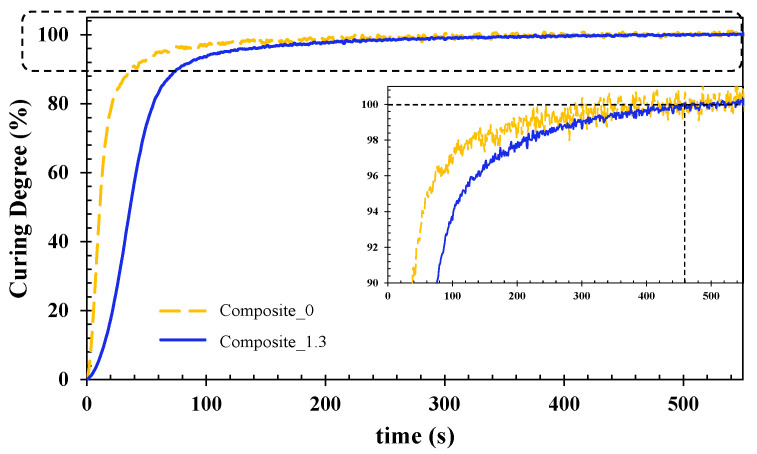
Curing degree evolution for unfilled composite (composite 0) and filled composite (composite 1.3) as a function of time 1 KHz.

Figure 5 compares the curing behavior of UV-cured unfilled composite (composite 0) and the fire-retardant composite with the ATH filler (composite 1.3). The inset was added to magnify the tendency of the curves from 90 to 100% curing degrees. 

The generated curves show the curing behavior of the UV-cured composites. Likewise, in the case of the resin formulations’ curing process, the dielectric signal (ionic viscosity) was monitored. The reaction took place through free radical polymerization, which involved the three steps of initiation, propagation, and termination which are represented by the rapid increase of the curves, the changes in the slope, and the final plateau, respectively. In this case, it is observed that there is a delay in the reaction behavior (the slope between 0 and 100 s) for the filled composite (composite 1.3). This difference is supported by the presence of ATH in composite 1.3, where other authors have reported that a high filler content leads to interfacial defects inside the material, thus provoking a delay in the reaction step [12]. Despite the difference at the beginning of the reaction for the unfilled composite (composite 0) and the filled composite (composite 1.3), similar curing times were reached at 460 s (see inset). Thus, it confirms the suitability of formulation 1.3 to manufacture glass-fiber-reinforced composites.

### 3.2. Mechanical Properties of Composite Parts

The obtained results from the flexural and interlaminar shear tests of the studied composites are detailed in Table 5. Composite 0, the unfilled composite, was considered as a reference and composite 1.3, the flame-retarded composite, was prepared with formulation 1.3.

Table 5 collects similar values for the unfilled (composite 0) and flame-retarded (composite 1.3) composites in terms of flexural strength. Although, between both of the composites, a difference of 5% in the fiber content is shown (unfilled (FVF, 30%)) and fire-retarded composite (FVF, 25%). However, the addition of inorganic flame-retardants in the latter case provides the stiffness to the final composite part, as represented in the modulus increase by 30%. As previously reported, the addition of inorganic fillers enhanced the mechanical properties of the polymer matrix composites [13]. In this case, to manufacture the flame-retarded composite, i.e., composite 1.3, the filler was selected as being the one which leads to an optimal viscosity. Due to its large particle size, it avoids the aggregation of particles and promotes its dispersion in the polymer matrix. Regarding the interlaminar shear strength (ILSS), both of the composites display similar values. Composite 1.3 results in a lower value, which is a consequence of the smaller resin percentage in the flame-retarded composite. A higher proportion of resin in the composite part favors the interlaminar strength of the final composite due to there being better interlaminar adhesion [14]. Therefore, the fire retardant additives have no negative effect on the mechanical properties.

### 3.3. Fire Behaviour of Composite Parts Test Methods

For the analysis and discussion of the results, only the classification parameters are taken into account. These are shown in Table 6, and they correspond to requirement R1 in the EN 45545-2 standard.

#### 3.3.1. Calorimetric Cone

**Figure 6:**
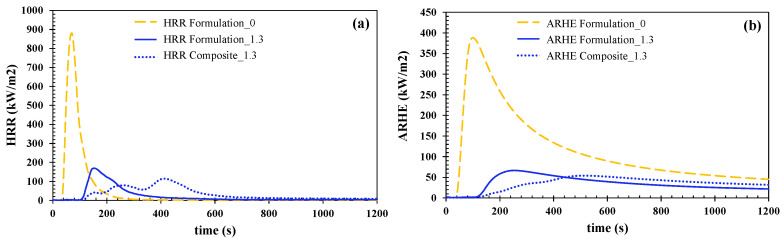
Heat Release Rate (HRR) (**a**) and Average Rate of Heat Emission (ARHE) (**b**) evolution over time for the tested formulations and composite as a function of time.

The three manufactured samples, formulation 0 was set as a reference sample, and formulation 1.3, the filled resin, and the glass-fiber-reinforced composite made from formulation 1.3 (i.e., composite 1.3) were tested according to the ISO 5660-1 standard. The irradiance that was used was 50 kW/m^2^, and the distance between the base of the cone and the surface of the specimen was 25 mm. Three specimens of each formulation were tested throughout the 20 min test. Figure 6 shows the average results that were obtained for cast-formed resin formulations and the flame-retarded composite.

The results for two parameters, HRR (a) and ARHE (b), are shown in Figure 6. The heat release rate is one of the most important parameters since an increase in the HRR translates into an increase in the thermal energy. This increase can favor flame propagation in the material [12]. Furthermore, by analyzing the curves (Figure 6a), it is possible to identify the different stages that occur in a combustion process: (i) the heating and the initiation of decomposition that leads to the generation of flammable gases; (ii) ignition; (iii) flame evolution; (iv) flame extinction. Another parameter that should be taken into account is the ignition time, which defines the ability of the material to withstand the heat flow before continuous combustion occurs [13]. For the flame-retarded resin (formulation 1.3), the ignition time leads to a raise of up to 66%, and for the flame-retarded composite (composite 1.3), an increase of 69% can be observed when it is compared to the pure resin (formulation 0). This small difference between the flame-retarded formulation and the composite is due to the major effect of the flame-retardant ATH in comparison to the contribution of the inert glass fiber. After this first period, a significant peak in the HRR is observed, which is attributed to the combustion of the flammable gases. Next, the HRR value gradually decreases due to the formation of a protective layer that slows down the decomposition of the material [14]. Finally, the HRR becomes negligible when the resin matrix decomposes completely. It can be noticed that in composite 1.3, the flame evolution step occurs in stages, which is in line with the decomposition of every different layer forming the composite.

On the other hand, Figure 6b shows the Average Rate of Heat Emission (ARHE), where its maximum (MARHE) is the most important parameter to take into account for classification purposes.

**Figure 7:**
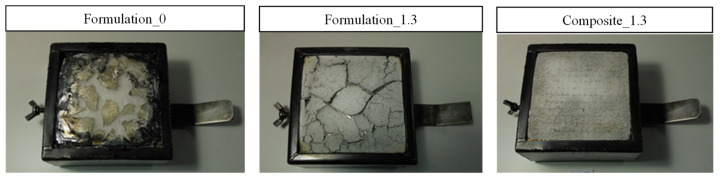
Representative examples of specimens after calorimetric cone experiments.

Figure 7 shows pictures of the surface of the studied specimens after the cone calorimeter test. It can be observed that for the unfilled resin (formulation 0), the specimen completely disappeared after the test. On the other hand, the flame-retarded resin formulation (formulation 1.3) presents cracks in the aluminum oxide protective layer which were formed by the ATH decomposition. Concerning the flame-retarded composite 1.3, the integrity of the sample remains, i.e., no cracks or holes can be observed. For the flame-retarded samples, formulation 1.3 and composite 1.3, a protective layer has been formed due to the ATH decomposition. This fact has been reported to be an effective method of improving the fire behavior of materials [15].

#### 3.3.2. Smoke Density and Gas Toxicity

Both the cast-formed unfilled and flame-retarded resin formulations, formulation 0 and formulation 1.3, respectively, and flame-retarded composite made from formulation 1.3 (composite 1.3) were tested to study the smoke density according to the ISO 5659-2 standard, and gas toxicity under EN 45545-2:2013 + A1:2015, Annex C. Method 1. 

Three specimens of each formulation were tested. The level of radiant heating used was 50 kW/m^2^ and the distance between the base of the cone and the surface of the specimen was 25 mm. Tests last 20 min although for classification purposes maximum density during the first 10 min must be collected.

Table 7 shows the average results that were obtained for cast-formed resin formulations (formulations 0 and 1.3) and the glass-reinforced composite made of formulation 1.3.

In the case of the cast-formed resin formulations, the value of Ds at the 4th minute is between 150 and 300 which implies that formulation 1.3 could aspire to an HL2 classification regarding the R1 requirement in the EN 45545-2 standard. On the one hand, concerning the glass-fiber-reinforced composites, this value is lower than 150, which implies a possible R1HL3 final classification. On the other hand, the VOF_4_ parameter is lower than 300 min for the ATH-filled systems, which implies that both of these systems fall within the R1HL3 classification range. In addition, the CIT_G_ values at the 4th and 8th minutes are also below 0.75, which also implies a possible R1HL3 classification for resin formulation 1.3 and composite 1.3.

To conclude, regarding the smoke density and gas toxicity parameters that were obtained, composite 1.3 could achieve R1HL3 as it meets all of the parameters demanded by this classification, while cast-formed resin formulation 1.3 would achieve an R1HL2 classification.

**Figure 8:**
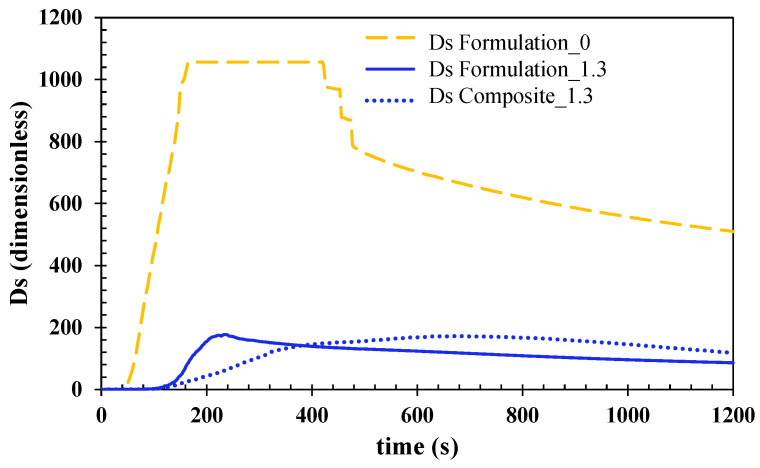
Smoke optical density (Ds) evolution over time of tested cast formulations and composite. One thousand and fifty-six is the maximum value that was measurable using the equipment.

Figure 8 shows the evolution of the optical density (dimensionless) of the materials that were tested over the 20-min test. The mean values of the three specimens that were tested for each of the three products that were analyzed are plotted. Formulation 0 has been represented as a reference to compare with it the flame-retarded formulation 1.3 and composite 1.3. The results show that specimens doped with ATH reduce, to a great extent, the optical smoke density of the material. This smoke reduction can be attributed to the formation of the Al_2_O_3_ protective layer which prevents a further reaction during the burning process, and consequently, decreases the release of smoke and the diluting effect of the H_2_O that is emitted during the ATH flame-retardant decomposition.

**Figure 9:**
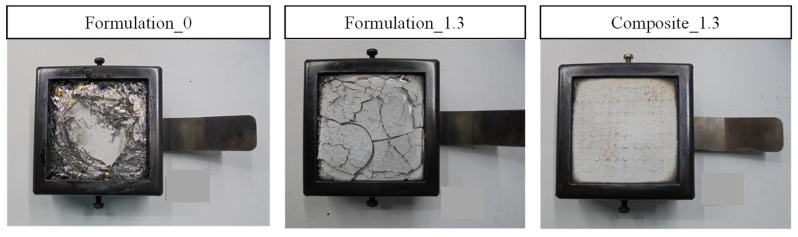
Representative examples for each formulation after smoke density and gas toxicity experiments.

Figure 9 shows pictures of the surface of the studied specimens after the smoke density and gas toxicity experiments. In the case of formulation 0, the pure resin sample, it is observed that there was a mainly total decomposition, whereas, for formulation 1.3 and composite 1.3, they were similar in the cone calorimeter experiments, and the addition of ATH prevented the samples from being decomposed. Apart from the fact that the Al_2_O_3_ layer generated is favorable for resisting the burning process, the water emitted from ATH during its decomposition dilutes the concentration of dense smoke and toxic gases [16].

#### 3.3.3. Lateral Flame Propagation

Three glass-fiber-reinforced composite 1.3 specimens were tested according to the ISO 5658-2 standard. Table 8 shows the average results that were obtained.

The parameter CFE is higher than 20 kW/m^2^, and so composite 1.3 could achieve an HL3 classification regarding R1 requirement that is contained in the EN 45545 standard.

In the picture that is shown above, one can observe three different areas after the flame propagation test from left to right. There is a calcinated zone where the resin has disappeared, another carbonized area where part of the resin remains, and a virgin one where the resin has not been visually affected. This fact is due to the different irradiances that were received by the test specimen along its length.

## 4. Conclusions

In this research, the effect of the flame-retardant ATH on a UV-curable thermosetting glass-fiber-reinforced composite was studied. A series of flame-retardant fillers were selected to analyze their behavior under the UV light curing method, which resulted in the suitability of Apyral20X for being incorporated in the UV resin formulations as well as in the subsequent manufactured flame-retarded composite. The following conclusions can be drawn from the performed study:

- The UV light transmission during the curing process is moderately affected by the nature of the ATH. As the specific surface area of the ATH becomes higher, longer times are needed for curing the specimens. The ATH flame retardants with the lowest specific surface area (Apyral20X) resulted in an invariant behavior despite the amount of ATH that was used in the resin mixture.
- Several UV flame-retarded resin formulations were developed, and the flame-retarded glass fiber reinforced composites were manufactured by the optimal one containing high amounts of ATH. Consequently, an increase in the viscosity of the formulation is observed. Therefore, to achieve a suitable impregnation of the glass fibers, the resin formulation is to be heated up to 60 °C to obtain a suitable processing technique.
- The composite that was developed shows the following behaviors: a moderate superficial flame spread, it does not generate flaming droplets/particles, the average rate of heat emission is low, and the emission of dense smoke and toxic gases is also quite moderate.
- Considering the results that have been obtained in the three individual tests that were carried out, and in classification terms, it can be concluded that the developed composite reaches the R1HL3 classifications according to the EN 45545-2 standard. This conclusion means that it can be used in large vehicle parts for indoor applications and in any type of railway vehicle.
- In addition, the developed composite also reaches the R7HL3 classifications according to the EN 45545-2 standard. This conclusion means that it can also be used in large vehicle parts for exterior applications and in any type of railway vehicle.

The settling of the flame-retardants over time in the polymeric matrix, when a high filler content is used, needs to be considered in a future study.

## Figures and Tables

**Table 1:** Flame-retardant fillers features.

Product	Particle Size D90 (μm)	BET (m^2^/g)	Oil Absorption (mL/100 g)
Apyral 20X	80	1.2	12
Apyral 30X	45	1.5	13
Apyral 22	40	2	13
Apyral 33	20	3	15

**Table 2:** Resin/filler blends prepared formulations.

Product	Reference	ATH (phr)
UV Resin	Formulation_0	0
Apyral20X	Formulation_1.1	20
	Formulation_1.2	40
	Formulation_1.3	70
Apyral30X	Formulation_2.1	20
	Formulation_2.2	40
	Formulation_2.3	70
Apyral22	Formulation_3.1	20
	Formulation_3.2	40
	Formulation_3.3	70
Apyral 33	Formulation_4.1	20
	Formulation_4.2	40
	Formulation_4.3	70

**Table 3:** Plateau values for different formulations of UV resin and fire-retardant filler as a function of grade and content of fillers. The standard deviation of four samples for each resin and filler blend.

ATH (phr)	Formulation_1	Formulation_2	Formulation_3	Formulation_4
0	414.27 ± 1.54	414.28 ± 1.54	414.27 ± 1.54	414.27 ± 1.54
20	420.75 ± 5.12	419.63 ± 3.94	463.50 ± 9.00	833.25 ± 6.30
40	414.75 ± 6.18	490.50 ± 9.00	522.75 ± 8.26	949.50 ± 7.84
70	414.75 ± 0.86	601.50 ± 8.39	761.25 ± 2.87	1396.13 ± 12.93

**Table 4:** Composition of the manufactured composites.

Product	Reinforcement	Matrix	FVF (%)	ATH (Apyral 20X) (phr)
Composite 0	MAT225/300/450/COMBI500	Formulation_0	29.48 ± 0.34	0
Composite 1.3		Formulation_1.3	24.45 ± 0.45	53.49 ± 0.43

**Table 5:** Mechanical properties of different composite formulations.

Property	Composite 0	Composite 1.3
Flexural Modulus, (MPa)	6010.00 ± 197.48	8720.33 ± 801.65
Flexural Strength, (MPa)	175.00 ± 3.92	183.95 ± 19.00
Interlaminar Shear Strength (ILSS), (MPa)	11.23 ± 0.67	10.66 ± 0.65
Fiber Volume Fraction, FVF (%)	29.48 ± 0.34	24.45 ± 0.45
Resin content (%)	70.52 ± 0.48	22.93 ± 0.32
ATH (phr)	0	53.49 ± 0.43

**Table 6:** Parameters and maximum and minimum values for R1 of the EN 45545-2 standard.

Requirement Set (Relevant Product No.)	Test Method Reference	Parameter and Unit	Maximum or Minimum	HL1	HL2	HL3
R1 (IN1A; IN1B; IN1D; IN1E; IN4; IN5; IN6A; IN7; IN8; IN9B; IN11; IN12A; IN12B; IN14; F5)	T02 ISO 5658-2	CFE, kW/m^2^	Minimum	20	20	20
	T03.01 ISO 5660-1: 50 kW/m^2^	MARHE, kW/m^2^	Maximum	-	90	60
	T10.01 EN ISO 5659-2: 50 kW/m^2^	D_s_(4), dimensionless	Maximum	600	300	150
	T10.02 EN ISO 5659-2: 50 kW/m^2^	VOF_4_, min	Maximum	1200	600	300
	T11. 01 EN ISO 5659-2: 50 kW/m^2^	CIT_G_, dimensionless	Maximum	1.2	0.9	0.75

**Table 7:** Smoke density and gas toxicity results of resin formulations (casts) and composite *.

Reference	Ignition Time (s)	Extinction Time (s)	Ds Minute 4	VOF_4_ (min)	D_S_ max 10 min	CIT_G_ 4 min	CIT_G_ 8 min
Formulation 0	43 ± 3	561 ± 97	1056.0 ± 0.0	2289.2 ± 42.8	1056.0 ± 0.0	0.160± 0.05	0.180 ± 0.02
Formulation 1.3	133 ± 6	506 ± 71	175.8 ± 23.5	218.7 ± 50.6	178.0 ± 22.9	0.040 ± 0.01	0.070 ± 0.01
Composite 1.3	-	-	65.4 ± 6.8	70.2 ± 5.7	175.7 ± 11.0	0.043 ± 0.003	0.101 ± 0.007

* Composite sample did not ignite, so there are no ignition and extinction time results.

**Table 8:** Lateral flame propagation of composite 1.3 and images of tested specimens.

Composite 1.3		
**Ignition time (s)**	142 ± 47	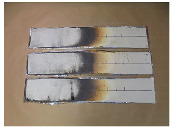
Extinction time (s)	880 ± 185	
CFE (kW/m^2^)	30.8 ± 6.4	
Maximum distance traveled (mm)	300.0 ± 50.0	
Flaming droplets and/or particles	NO	
